# A retrospective matched cohort study evaluating the effects of percutaneous endoscopic gastrostomy feeding tubes on nutritional status and survival in patients with advanced gastroesophageal malignancies undergoing systemic anti-cancer therapy

**DOI:** 10.1371/journal.pone.0188628

**Published:** 2017-11-29

**Authors:** Scott Mitchell, John P. Williams, Harsimrandeep Bhatti, Toufic Kachaamy, Jeffrey Weber, Glen J. Weiss

**Affiliations:** 1 Arizona State University, Tempe, Arizona, United States of America; 2 Western Regional Medical Center, Cancer Treatment Centers of America, Goodyear, Arizona, United States of America; University Hospital Llandough, UNITED KINGDOM

## Abstract

**Background:**

Many patients with cancer or other systemic illnesses can experience malnutrition. One way to mitigate malnutrition is by insertion of a percutaneous endoscopic gastrostomy feeding tube (PEG tube). The goal of this retrospective matched cohort study is to evaluate if PEG tube placement improved nutritional status and overall survival (OS) in advanced gastroesophageal (GE) cancer patients who are undergoing anti-neoplastic therapy.

**Methods:**

GE cancer patients who were treated and evaluated by a nutritionist and had at least 2 nutritionist follow-up visits were identified. Patients with PEG tube were matched to patients that did not undergo PEG placement (non-PEG). Clinical characteristics, GE symptoms reported at nutrition follow-up visits, and OS were recorded.

**Results:**

20 PEG and 18 non-PEG cases met criteria for further analyses. After correction for multiple testing, there were no OS differences between PEG and non-PEG, treatment naive and previously treated. However, PEG esophageal carcinoma has statistically significant inferior OS compared with non-PEG esophageal carcinoma. PEG placement did not significantly reduce the proportion of patients with weight loss between the initial nutrition assessment and 12-week follow-up.

**Conclusions:**

In this small study, PEG placement had inferior OS outcome for GE esophageal carcinoma, no improvement in OS for other evaluated groups, and did not reduce weight loss between baseline and 12-week follow-up. Unless there is prospective randomized trial that can show superiority of PEG placement in this population, PEG placement in this group cannot be endorsed.

## Introduction

Gastroesophageal (GE) cancers are known to commonly produce a variety of GE-related symptoms, such as dysphagia and cachexia that can lead to malnutrition [1]. The prevalence of malnutrition incidents in these populations can range from 40–85% of patients [1,2]. Controlling malnutrition is a major concern in the treatment of patients undergoing anti-neoplastic therapy. Cancer-associated malnutrition has been shown to lower a patient’s response to anti-neoplastic therapies, as well as, amplify the risk of chemotherapy-induced toxicity [3]. In addition, malnutrition is one of the main contributors towards a decline in a patient’s nutritional status and their overall quality of life (QoL) [4]. Malnutrition has also been shown to lead to an increase in health care costs [5]. Thus, mitigating and alleviating malnutrition is a major concern in the management of cancer patients receiving anti-neoplastic therapies.

One way to remedy malnutrition is through the use of a percutaneous endoscopic gastrostomy feeding tube (PEG tube). PEG tubes are inserted into the stomach, usually through abdominal wall, and are used to provide enteral nutrition to patients in which the oral cavity must be bypassed [6]. While previous studies have shown a a positive correlation between nutritional stasis and overall QoL, the relationship between PEG tube use, nutrition status, and treatment outcomes in patients who are undergoing anti-neoplastic therapies has not been thoroughly examined [7–10].

The goal of this retrospective matched cohort study is to evaluate if PEG tube placement improved nutritional status and overall survival (OS) in advanced GE cancer patients who are undergoing anti-neoplastic therapy.

## Materials and methods

### Ethics statement

The study was conducted based on the ethical standards prescribed by the Helsinki Declaration of the World Medical Association and with the approval of the Western Institutional Review Board (WIRB) study #20140710. As this was a retrospective study of existing data, the requirement for patient consent was waived by WIRB and consent was not obtained for this study. Clinical information was collected, and patients were de-identified by removing all identifiers that could be linked back to the patient.

### Study design

We identified GE cancer patients with metastatic disease that received oncology care at a single comprehensive cancer center and were evaluated by a nutritionist, between December 2008 to February 2014 and had at least 2 nutritionist follow-up visits. The first follow-up visit was between 4–6 weeks and the second visit was approximately at 12 weeks from the initial nutrition assessment. Patients with PEG tube were matched by histology type, similar age range (+/- 5 years), treated during a similar time frame (within ~12 months of each other) to patients that did not undergo PEG placement (non-PEG).

### Study endpoints

#### Statistical analysis

Age, gender tumor histology, stage at initial assessment at the cancer center, weight change, and the symptoms nausea, diarrhea, constipation, taste changes, sensitivity to smells, dysphagia, odynophagia, anorexia, cachexia, edema, and anemia were measured at baseline, once between 4–6 weeks and then at 12 weeks from the baseline assessment. Type of prior therapy and OS from time of initial nutritionist consultation were recorded as well. To determine the nutritional status of the patients, the weight loss percentages of patients over the course of 12 weeks were recorded and chi square statistics were calculated to determine if the findings were statistically significant. For OS, Kaplan-Meier survival curves were constructed under specific perimeters. Two-tailed p-values less than 0.05 were considered statistically significant. For multiple tests evaluating OS, Bonferroni correction was applied and a p-value less than 0.010 was considered statistically significant.

## Results

### Patient characteristics

**Fig 1:**
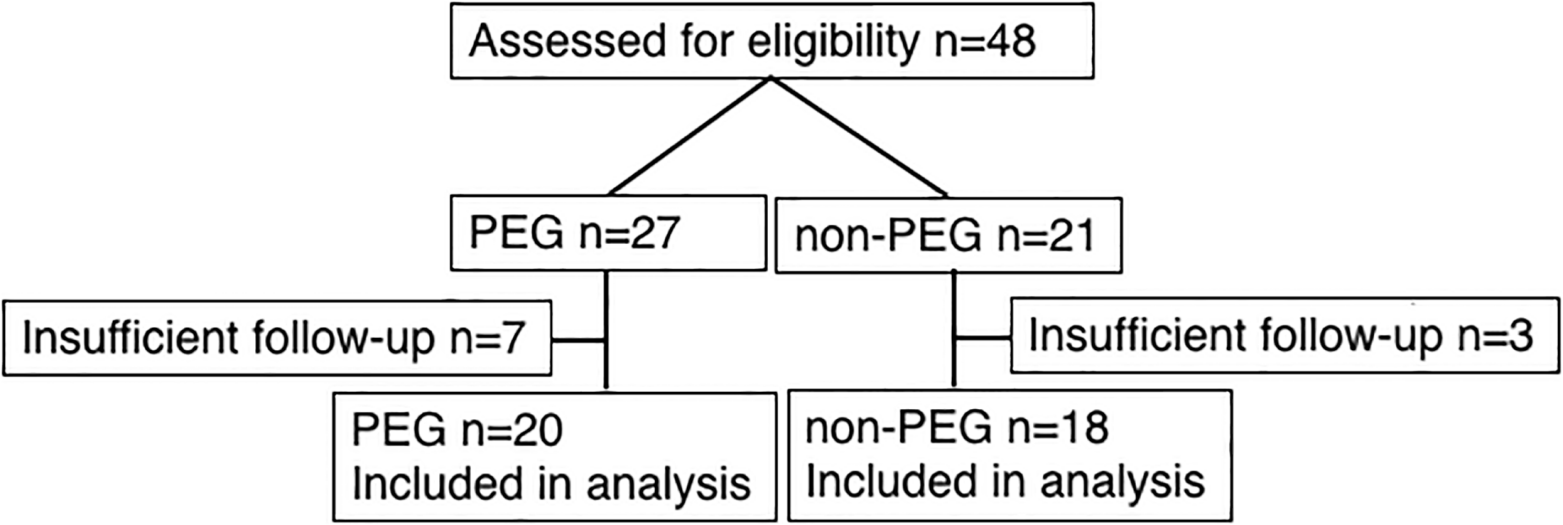
CONSORT diagram. CONSORT diagram depicting number of patients evaluated for eligibility and number of patients included in analysis.

Initially there were 27 GE patients identified with PEG tube placement, and another 21 GE patients that did not have PEG tube placement during this time frame that were available for cohort matching (non-PEG). Upon further review of records, 10 patients, seven PEG and 3 non-PEG, were considered not evaluable due to insufficient follow-up (Fig 1 and S1 Table).

Of those patients with PEG tube placement, 16 had adenocarcinoma histology, 9 had squamous cell carcinoma (SCC), and 2 had neuroendocrine tumors. Of those non-PEG patients identified, 11 had adenocarcinoma histology, 9 had SCC, and 1 had neuroendocrine histology (Table 1). PEG patients were significantly less likely to be treatment-naive (p = 0.019) and had a higher prevalence of esophageal cancer (p = 0.023).

**Table 1:** Clinical characteristics.

Clinical Characteristics	PEG n = 20	Non-PEG n = 18
**Median age (range)**	58 (30–66)	55 (35–63)
**Gender**	Male 19/ Female 1	Male 17/ Female 1
**Esophageal cancer**	16	8
**Gastroesophageal junction cancer**	4	10
**Percentage weight loss at baseline nutrition assessment**	>4.9%: 11, <5.0%: 9	>4.9%: 10, <5.0%: 8
**Treatment-naive prior to nutrition assessment**	26.7% (n = 15)	68.8%(n = 16)

Tables 2 and 3 depict the symptoms of PEG and non-PEG patients recorded at baseline and the follow-up nutritional assessments, respectively. The PEG tube population reported a decrease in anorexia and nausea but an increase in dysguesia between baseline and the 12-week nutritional assessment. The non-PEG tube population reported lower decreased appetite, dysphagia, and cachexia during this interval.

**Table 2:** Symptoms of PEG patients (n = 20).

	Nausea	Diarrhea	Constipation	Dysguesia	Decreased appetite	Dysphagia	Odynophagia	Edema	Anorexia	Cachexia	Anemia
**Baseline (%)**	9 (45)	5 (25)	5 (25)	3 (15)	8 (40)	13 (65)	3 (15)	2 (10)	7 (35)	2 (10)	4 (20)
**4–6 Weeks (%)**	10 (50)	4 (20)	3 (15)	5 (25)	5 (25)	13 (65)	6 (30)	3 (15)	2 (10)	2 (10)	2 (10)
**12 Weeks (%)**	10 (50)	4 (20)	4 (20)	6 (30)	6 (30)	10 (50)	4 (20)	1 (5)	3 (0.15)	1 (5)	1 (5)

**Table 3:** Symptoms of non-PEG patients (n = 18).

	Nausea	Diarrhea	Constipation	Dysguesia	Decreased Appetite	Dysphagia	Odynophagia	Edema	Anorexia	Cachexia	Anemia
**Baseline (%)**	2 (11)	2 (11)	3 (17)	2 (11)	7 (39)	8 (44)	2 (11)	0	2 (11)	3 (17)	1 (6)
**4–6 Weeks (%)**	3 (17)	1 (6)	1 (6)	2 (11)	3 (17)	7 (39)	2 (11)	0	1 (6)	1 (6)	0
**12 Weeks (%)**	2 (11)	4 (22)	1 (6)	2 (11)	4 (22)	4 (22)	1 (6)	1 (6)	1 (6)	0	0

**Fig 2:**
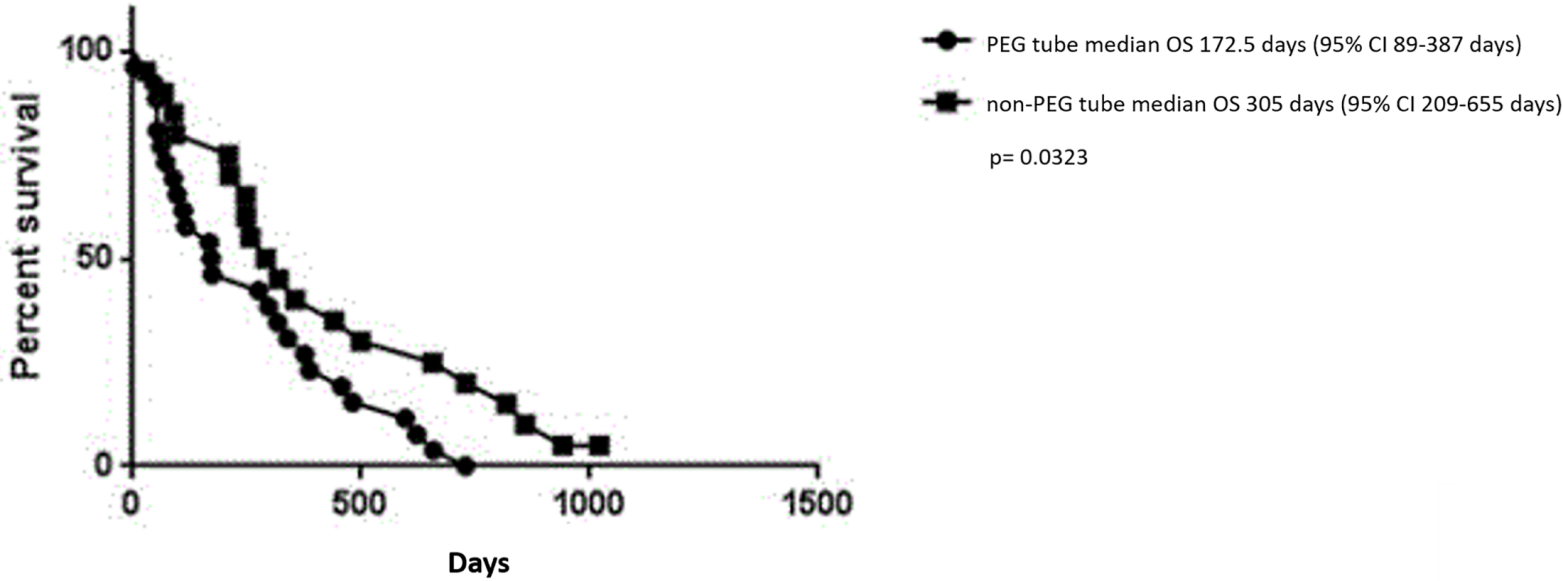
OS comparing PEG vs non-PEG in all patients. Kaplan-Meier curve depicting the estimated OS of all patients. The line with circles depicts PEG patients, while the line with squares depicts non-PEG patients. P-value is not significant after Bonferroni correction.

Fig 2 shows the OS between patients receiving PEG tube placement and the matched non-PEG cohort (p = 0.0323), after correction for multiple testing this did not reach statistical significance.

**Fig 3:**
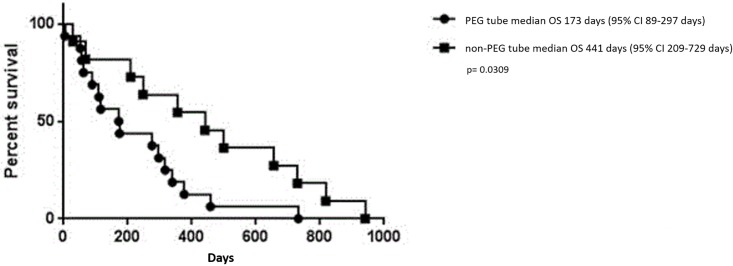
OS comparing PEG vs non-PEG in adenocarcinoma. Kaplan-Meier curve depicting the estimated OS for patients with adenocarcinoma. The line with circles depicts PEG adenocarcinoma patients, while the line with squares depicts non-PEG adenocarcinoma patients. P-value is not significant after Bonferroni correction.

Fig 3 shows the OS differences between PEG adenocarcinoma patients and the matched non-PEG adenocarcinoma cohort (p = 0.0309), after correction for multiple testing this did not reach statistical significance.

**Fig 4:**
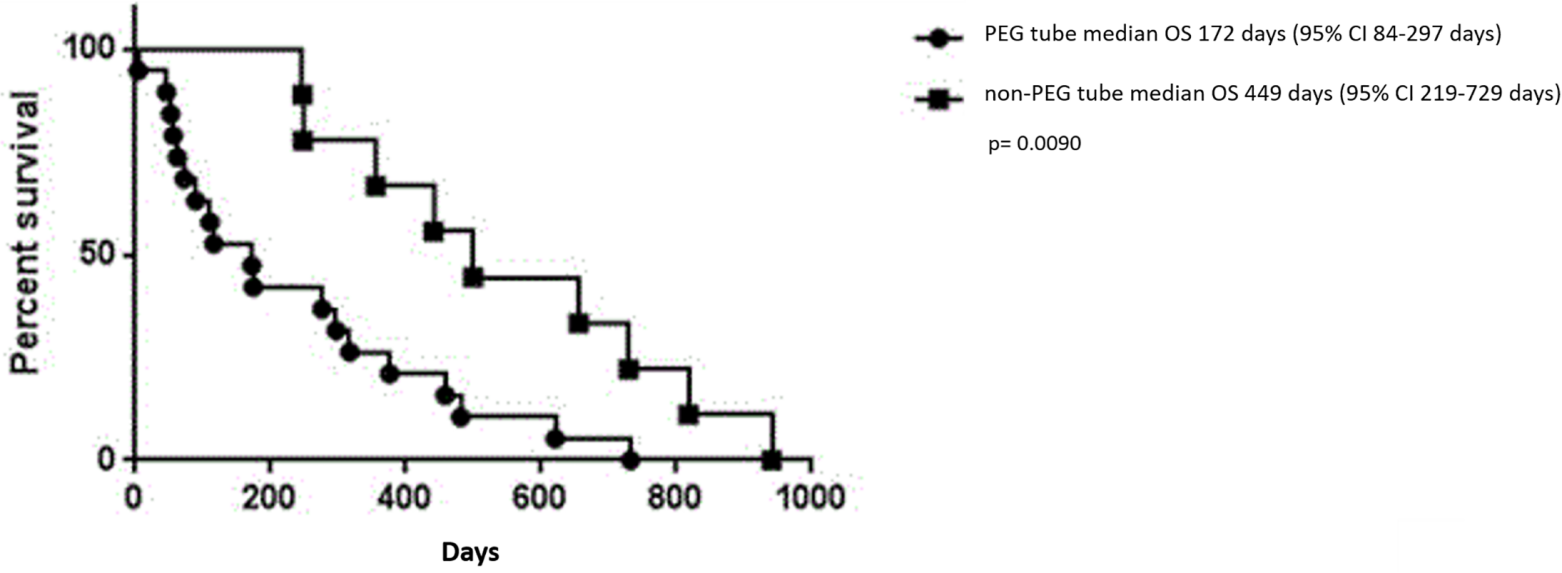
OS comparing PEG vs non-PEG in esophageal carcinoma. Kaplan-Meier curve depicting the estimated OS for patients with esophageal carcinoma. The line with circles depicts PEG esophageal carcinoma patients, while the line with squares depicts non-PEG esophageal carcinoma patients. P-value is significant after Bonferroni correction.

The survival difference is significantly more pronounced between PEG tube and non-PEG esophageal carcinoma patients (p = 0.0090), favoring non-PEG (Fig 4).

In contrast to adenocarcinoma, for SCC there is no OS difference between PEG tube and non-PEG tube patients (p = 0.8181) (S1 Fig).

Receipt of prior systemic therapy does not appear to influence OS in patients (p = 0.6179) (S2 Fig).

PEG placement did not significantly reduce the proportion of patients with weight loss between the initial nutrition assessment and 12-week follow-up (S2 and S3 Tables). For the overall population, there was a non-statistically significant increase in the proportion of patients with < 4.9% weight loss by the 12-week follow-up (S4 Table).

## Discussion

The OS for patients with metastatic GE cancers is poor. The prognosis is worse for those experiencing GE-related symptoms, especially malnutrition. The cause of the malnutrition is primarily due to the cancer and overall tumor burden. If a systemic therapy is appropriate and successful in significantly decreasing tumor burden, patients can experience an improvement in QoL, including nutrition status [8]. The ability to mitigate malnutrition by proactive measures in an effort to optimize the patient to receive systemic therapy are sought. Several options exist including nutrition counseling and interventions such as PEG tube placement. The relationship between PEG tube placement, nutrition status, and OS outcomes in metastatic GE cancer patients undergoing systemic therapy has not been well-examined.

The main finding from our retrospective case matched analysis is that the OS for PEG tube esophageal carcinoma population was significantly worse than the non-PEG esophageal carcinoma population (Fig 4). This result was surprising. As clinicians, we believe that for those patients unable to maintain adequate nutrition, that it would be logical that this intervention would help sustain and nutritionally optimize a patient to receive and continue with systemic therapy. Due to the small numbers in this retrospective analysis and multiple testing, we were not able to show overall that PEG compared with non-PEG had statistically different OS. There did not appear to be a difference in OS in SCC patients or those who received prior systemic therapy at the time of initial nutrition assessment.

The non-PEG tube population reported lower decreased appetite, dysphagia, and cachexia at the 12-week assessment compared to the initial nutrition assessment. The PEG tube population reported a decrease in anorexia and nausea but an increase in dysguesia during this interval. Overall, both PEG and non-PEG populations did not have any statistically significant weight loss changes at the 12-week nutrition assessment.

There are a few limitations associated with this study. The study was retrospective and the sample size was small. Retrospective studies have recorded limitations themselves such as difficulty in determining a cause and effect relationship, as well as, relying on past recordings of data [1]. Small sample sizes often cause outlier data to more profound, which can skew the overall results. Even though this study was limited to GE cancers, systemic treatments vary somewhat and evaluation of patients were not uniform in their disease spectrum. Some patients were treatment naïve while others had prior systemic therapy elsewhere before being evaluated at the cancer center. Overall, the two groups were well matched. The PEG group did have a significantly higher proportion of patients with esophageal cancer and patients that had prior treatment before the baseline nutritionist evaluation, which could impact OS. However, S2 Fig, shows similar OS curves between treatment-naive and previously treated patients. Despite these limiting factors, we still observed a significant difference in OS in GE esophageal carcinoma patients favoring non-PEG after Bonferroni correction. We also acknowledge that this study has some limitations with regards power, selection bias, design, and indication bias, however, the current realities of clinical practice impede a prospective randomized trial from being carried out practically without some supporting information to justify the time, expense, and effort. We hope that our findings will foster sufficient interest to attempt to prove or disprove the benefit of PEG in cancer patients. Unless there is prospective randomized trial that can demonstrate superiority of PEG placement in this population, PEG placement in this group cannot be endorsed.

## Supporting information

S1 Fig OS comparing PEG vs non-PEG in SCC. Kaplan-Meier curve depicting the estimated OS for SCC patients. The line with circles depicts PEG SCC patients, while the line with squares depicts non-PEG SCC patients. P-value is not significant after Bonferroni correction.
(TIF)

S2 Fig OS difference between systemic therapy naive vs previously treated patients prior to initial nutrition assessment. Kaplan-Meier curve depicting the estimated OS for patients with and without prior systemic therapy at the time of initial nutrition assessment. The line with circles depicts patients that received prior systemic therapy at the time of initial nutrition assessment, while the line with squares depicts systemic therapy naïve patients at the time of initial nutrition assessment. P-value is not significant after Bonferroni correction.
(TIF)

S1 Table De-identified dataset. This is the dataset for all 48 identified patients.
(XLSX)

S2 Table Comparison of PEG vs. non-PEG patients with less than 4.9% weight loss between initial nutrition assessment and 12-week follow-up. Number of cases of PEG vs. non-PEG with less than 4.9% weight loss between these time points.
(DOCX)

S3 Table Comparison of PEG vs. non-PEG patients with greater than 5% weight loss between initial nutrition assessment and 12-week follow-up. Number of cases of PEG vs. non-PEG with more than 5% weight loss between these time points.
(DOCX)

S4 Table Comparison of weight loss changes by less than 4.9% vs greater than 5% between initial nutrition assessment and 12-week follow-up. Number of cases of with weight loss less than 4.9% vs. weight loss more than 5% between these time points.
(DOCX)
