# Supplementary figures and images for: A retrospective matched cohort study evaluating the effects of percutaneous endoscopic gastrostomy feeding tubes on nutritional status and survival in patients with advanced gastroesophageal malignancies undergoing systemic anti-cancer therapy
Source: PLoS One. 2017 Nov 29;12(11):e0188628. doi: 10.1371/journal.pone.0188628 (PMC5706679; doi:10.1371/journal.pone.0188628)

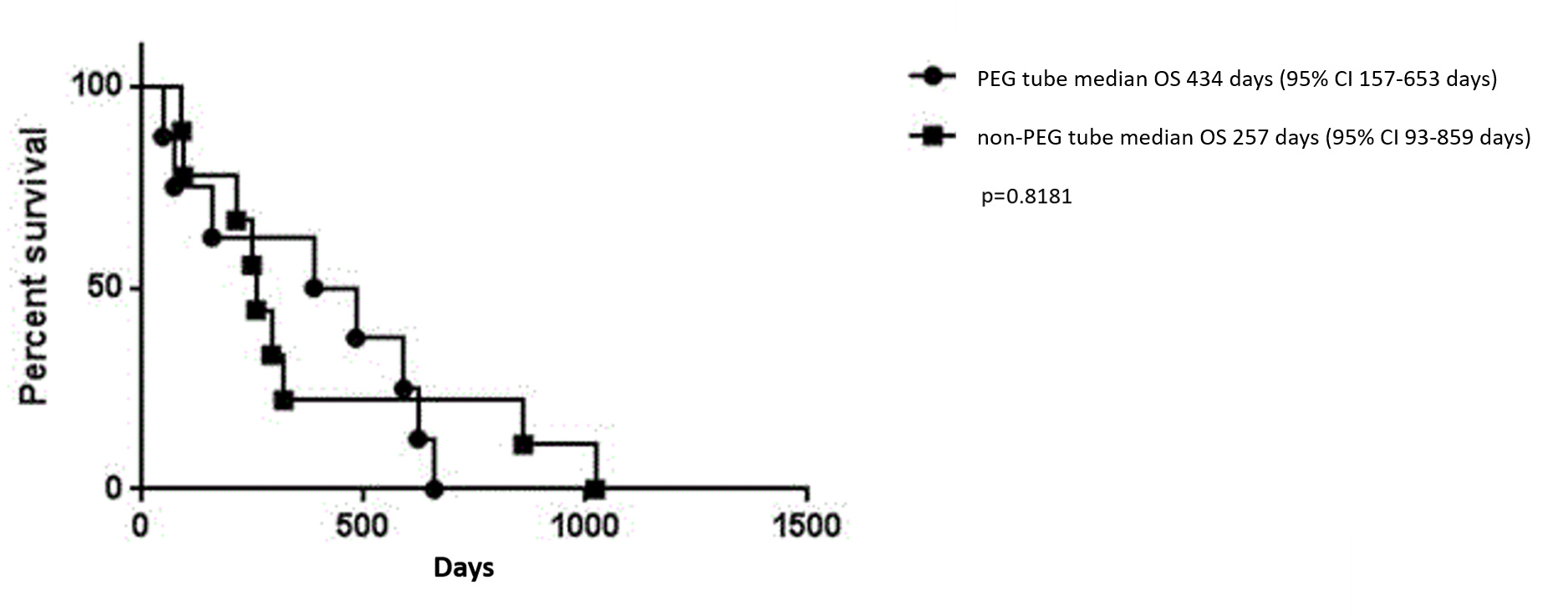

Supplement: S1 Fig — Kaplan-Meier curve depicting the estimated OS for SCC patients. The line with circles depicts PEG SCC patients, while the line with squares depicts non-PEG SCC patients. P-value is not significant after Bonferroni correction. (TIF) [file pone.0188628.s001.tif]

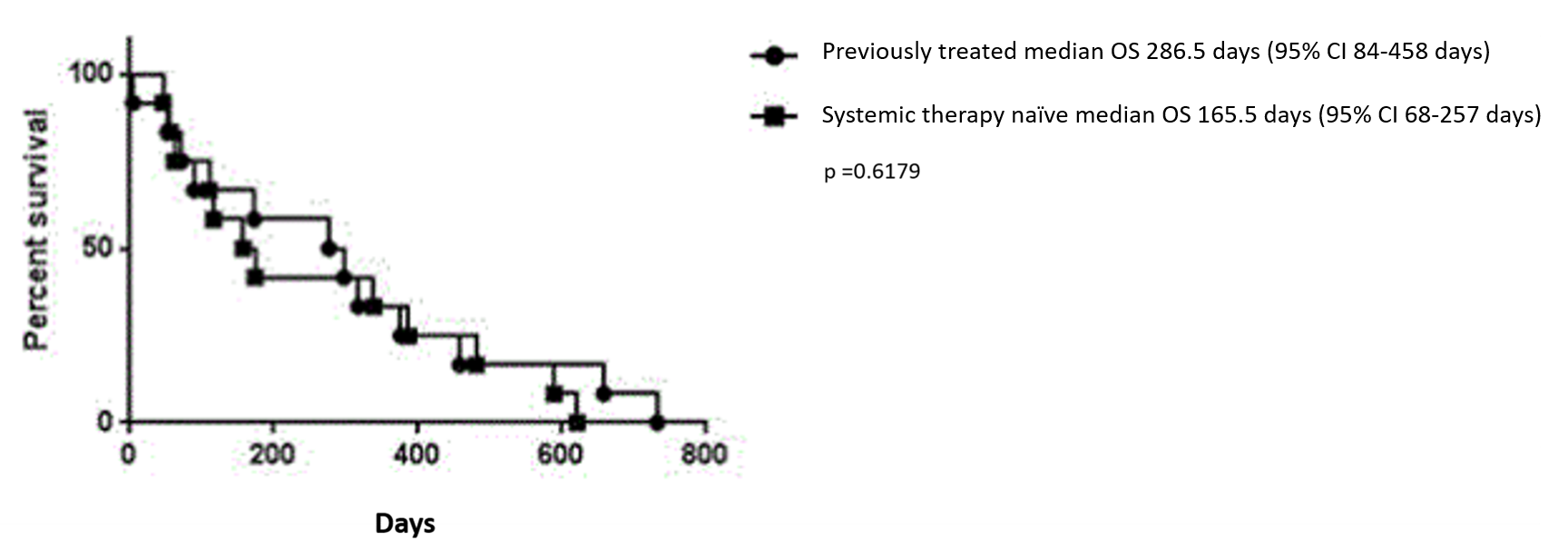

Supplement: S2 Fig — Kaplan-Meier curve depicting the estimated OS for patients with and without prior systemic therapy at the time of initial nutrition assessment. The line with circles depicts patients that received prior systemic therapy at the time of initial nutrition assessment, while the line with squares depicts systemic therapy naïve patients at the time of initial nutrition assessment. P-value is not significant after Bonferroni correction. (TIF) [file pone.0188628.s002.tif]
