# Supplementary material for: A retrospective matched cohort study evaluating the effects of percutaneous endoscopic gastrostomy feeding tubes on nutritional status and survival in patients with advanced gastroesophageal malignancies undergoing systemic anti-cancer therapy
Source: PLoS One. 2017 Nov 29;12(11):e0188628. doi: 10.1371/journal.pone.0188628 (PMC5706679; doi:10.1371/journal.pone.0188628)
Supplement: S2 Table — Number of cases of PEG vs. non-PEG with less than 4.9% weight loss between these time points. (DOCX) [file pone.0188628.s004.docx]

**S2 Table: Comparison of PEG vs. non-PEG patients with less than 4.9% weight loss between initial nutrition assessment and 12-week follow-up.**

|  | PEG | non-PEG |
| --- | --- | --- |
| Baseline | 11 | 10 |
| 12 Weeks | 15 | 16 |

There was no significant difference between PEG and non-PEG.
