# Supplementary material for: A retrospective matched cohort study evaluating the effects of percutaneous endoscopic gastrostomy feeding tubes on nutritional status and survival in patients with advanced gastroesophageal malignancies undergoing systemic anti-cancer therapy
Source: PLoS One. 2017 Nov 29;12(11):e0188628. doi: 10.1371/journal.pone.0188628 (PMC5706679; doi:10.1371/journal.pone.0188628)
Supplement: S4 Table — Number of cases of with weight loss less than 4.9% vs. weight loss more than 5% between these time points. (DOCX) [file pone.0188628.s006.docx]

**S4 Table:** **Comparison of weight loss changes by less than 4.9% vs greater than 5% between initial nutrition assessment and 12-week follow-up.**

|  | < 4.9% | > 5.0% |
| --- | --- | --- |
| Baseline | 19 | 13 |
| 12 Weeks | 26 | 6 |

There was no significant difference between <4.9% and >5.0%.
